# Machining-Induced Work Hardening Behavior of Inconel 718 Considering Edge Geometries

**DOI:** 10.3390/ma15020397

**Published:** 2022-01-06

**Authors:** Bin Zhou, Weiwei Zhang, Zhongmei Gao, Guoqiang Luo

**Affiliations:** 1 Hubei Digital Manufacturing Key Laboratory, School of Mechanical and Electronic Engineering, Wuhan University of Technology, Wuhan 430070, China; zhb_8250@163.com (B.Z.); zwwvivid@whut.edu.cn (W.Z.); 2 State Key Laboratory of Digital Manufacturing Equipment and Technology, Huazhong University of Science and Technology, Wuhan 430074, China; 3 Chaozhou Branch of Chemistry and Chemical Engineering Guangdong Laboratory (Hanjiang Laboratory), Chaozhou 521000, China; luogq@whut.edu.cn; 4 State Key Laboratory of Advanced Technology for Materials Synthesis and Processing, Wuhan University of Technology, Wuhan 430070, China

**Keywords:** Inconel 718, work hardening, CEL, chamfered edge

## Abstract

As a representative type of superalloy, Inconel 718 is widely employed in aerospace, marine and nuclear industries. The significant work hardening behavior of Inconel 718 can improve the service performance of components; nevertheless, it cause extreme difficulty in machining. This paper aims to investigate the influence of chamfered edge parameters on work hardening in orthogonal cutting of Inconel 718 based on a novel hybrid method, which integrates Coupled Eulerian-Lagrangian (CEL) method and grain-size-based functions considering the influence of grain size on microhardness. Orthogonal cutting experiments and nanoindentation tests are conducted to validate the effectiveness of the proposed method. The predicted results are highly consistent with the experimental results. The depth of work hardening layer increases with increasing chamfer angle and chamfer width, also with increasing feed rate (the uncut chip thickness). However, the maximum microhardness on the machined surface does not exhibit a significant difference. The proposed method can provide theoretical guidance for the optimization of cutting parameters and improvement of the work hardening.

## 1. Introduction

As a representative type of difficult-to-cut material, Inconel 718 is widely employed in aerospace, nuclear and marine industries for its superior physical properties. The increase of microhardness near the surface caused by machining, i.e., work hardening, can practically improve the service performance of parts while cause severe tool damage during processing [1]. It has been proven that Inconel 718 exhibits obvious work hardening behavior in the material removal process, causing severe damage to inserts and reducing tool life [2]. To investigate the work hardening behavior in metal cutting to improve the machinability of such material, many researches have been working to determine the machining-induced microhardness variation in subsurface area.

Most of the relevant studies focused on the effects of cutting parameters (e.g., feed rate, cutting velocity, depth of cut), tool geometry (e.g., rake angle, nose radius), or auxiliary methods (e.g., lubricant, coolant, laser-assisted machining) on work hardening. Ren and Liu [3] stated that increasing cutting speed could reduce the depth of affected area while improving the degree of work hardening when turning Inconel 718. A positive effect of cutting speed on the degree of work hardening was also found by Hua and Liu [4]. Meanwhile, their research presented that feed rate was a more significant factor whose growth can increase both the depth of affected area and the work hardening degree. The reduction of tool nose radius played the same role as the growth of feed rate, resulting in deeper work hardening depth and greater work hardening degree. Choi [5] carried out cutting experiments of AISI 1053 steel employing tools with different rake angles, and found that a softer machined layer can be obtained by tools with less positive rake angles. Shokrani et al. [6] studied the influence of cooling conditions on surface integrity of Ti-6Al-4V. The results showed that cryogenic machining produced the highest surface microhardness, and flood cooling induced the maximum depth of work hardening. Kalantari et al. [7] compared the performance of conventional and laser-assisted machining of titanium. It was found that the laser-assisted machining exhibited superior advantages in reducing the microhardness of subsurface area.

Apart from the above mentioned factors, edge geometry was validated as another essential reason that can change the cutting mechanisms and then affect the work hardening layer of components [8]. For honed edge, Arısoy et al. [9] investigated tools with three levels of edge radius (5 μm, 10 μm, 20 μm) in face turning of Inconel 100, and observed that the tools with the largest edge radius resulted in the highest microhardness level in subsurface area. Similar trends were also presented by Denkena et al. [10] in hard turning of AISI 52100 bearing steel and Li et al. [11] in hard milling of AISI H13 steel. For chamfered edge, Pawade et al. [12] employed three types of cutting edge in high speed turning of Inconel 718, that was, 30° chamfer angle with 100 μm chamfer width (CW1), 20° chamfer angle with 100 μm chamfer width (CW2), and 30° chamfer angle with 100 μm chamfer width plus hone (CH). The results showed that CW1 tool produced the highest degree of work hardening, while CH tool produced the lowest degree. Zhuang et al. [13] found that increasing chamfer width would cause greater degree of work hardening, while chamfer angle did not make noticeable difference. Tool wear represents another situation that edge geometry changes. Taking flank wear as an example, the major agreement in this field is that the longer the flank wear is, the more severe the work hardening phenomenon will be [14].

Experimental results only represent a limited law within the tested groups, serving for a specific tool–workpiece couple. With the requirements of functional design and deep understanding of work hardening behavior, predictive models are also developed, which can be classified as analytical, numerical, empirical, hybrid, etc. Hughes et al. [15] proposed Zener–Hollomon and Hall–Petch functions for predicting the grain size and the microhardness in subsurface, respectively. Inspired by their studies, Ren and Liu [16] attempted to use hybrid methods to evaluate work hardening in machining, which employed these functions as user subroutines in finite element simulations. Larger cutting speed and lower feed rate were found to obtain better surface quality when machining Inconel 718. A similar approach can be found in Ref [17], where enlarging the feed rate can increase the white layer while decrease the dark layer. Ding and Shin [18] developed a multi-physics model considering the influence of phase transformation and grain refinement for AISI 52100 steel, which also employed the physical variables given by simulations. Zhang et al. [19] proposed a fully analytical method for the prediction of work hardening behavior of AISI 304 stainless steel. The study found that larger cutting depth resulted in a greater gradient of microhardness near the surface, and the cutting speed slightly affected the subsurface microhardness distribution. In addition to these methods, Arısoy et al. [9] gave an empirical model considering the relation between work hardening and cutting parameters, which showed practical effectiveness for face turning of Inconel 100. Detailed information on the modeling of work hardening in metal cutting is given in Section 2.

This paper aims to investigate the influence of chamfered edge parameters on work hardening in orthogonal cutting of Inconel 718 based on a novel hybrid method, which integrates Coupled Eulerian-Lagrangian (CEL) method and grain-size-based functions considering the influence of grain size on microhardness. The orthogonal cutting process was simulated with different settings of chamfer angle, chamfer width and feed rate. For each case, Zener–Hollomon and Hall–Petch equations were employed as subroutines to determine the microhardness in the subsurface area. The effectiveness of the proposed method was validated by nanoindentation tests. Further, the effects of chamfer parameters and feed rate on work hardening behavior were discussed.

## 2. Literature Review of Microhardness Modeling in Machining

Metal cutting process represents severe plastic deformation caused by mechanical and thermal loads, leading to a series of cutting phenomena, including phase transformation, recrystallization, dislocation, etc. Modeling of work hardening layer induced by machining requires three parts: temperature history, stress-strain history and hardness modeling. For the temperature history, analytical methods usually employ the image heat source theory considering the temperature rise generated by shear zone heat source and rubbing heat source in the tool-workpiece interface [20]. The expressions can be illustrated as below:

(1) $$\left\{\begin{array}{l} \Delta T_{1}=\frac{q_{s}}{2\pi \lambda_{w}}\int_{0}^{L_{s}}\exp\left(\frac{V(x+l_{i}\cos f)}{2a_{w}}\right)\left[\begin{array}{l} K_{0}\left(\frac{V}{2a_{w}}\sqrt{{\left(x+l_{i}\cos f\right)}^{2}+{\left(z+l_{i}\sin f\right)}^{2}}\right)+\\ K_{0}\left(\frac{V}{2a_{w}}\sqrt{{\left(x+l_{i}\cos f\right)}^{2}+{\left(z+2t-l_{i}\sin f\right)}^{2}}\right) \end{array}\right]dl_{i}\\ \Delta T_{2}=\frac{q_{r}}{\pi \lambda_{w}}\int_{0}^{L_{r}}\left\{B_{w}\exp\left(\frac{V(x-l_{i})}{2a_{w}}\right)K_{0}\left(\frac{V}{2a_{w}}\sqrt{{\left(x-l_{i}\right)}^{2}+z^{2}}\right)\right\}dl_{i} \end{array}\right.$$

where Δ*T*_1_ and Δ*T*_2_ are the temperature rise in workpiece caused by heat source of the shear zone and tool-workpiece interface, respectively. *q*_s_ and *q*_r_ are the heat intensity of these two heat sources. *λ*_w_ and *a*_w_ are the thermal conductivity and the thermal diffusivity of workpiece material, respectively. *L*_s_ and *L*_r_ are the length of the shear zone and tool-workpiece interface, respectively. *B*_w_ is the heat partition transferred into workpiece. *V*, *t* and *ϕ* are the cutting velocity, uncut chip thickness and shear angle, respectively. *K*_0_ is Bessel function. *x* and *z* are the coordinates in the subsurface area. While using finite element method, the calculation of thermal equilibrium in explicit mode is always based on Newton–Raphson equation as follows [21]:

(2) $$C_{T}\dot{T}+K_{T}T=Q$$

where *T* is the temperature vector; *C*_T_ is the capacitance matrix; *K*_T_ is the heat conduction matrix; *Q* is the external and internal heat flux vector.

For the stress-strain history, analytical methods always include two steps. The first step is to model the stress field in the subsurface area considering stress components in a plane, given by Ref. [22].

(3) $$\left\{\begin{array}{l} \sigma_{xx}(x,z) =-\frac{2z}{\pi }\int_{-a}^{a}\frac{p_{n}(s)(x-s{)}^{2}ds}{{[(x-s{)}^{2}+z]}^{2}}-\frac{2}{\pi }\int_{-a}^{a}\frac{p_{t}(s)(x-s{)}^{3}ds}{{[(x-s{)}^{2}+z]}^{2}}\\ \sigma_{zz}(x,z) =-\frac{2z^{3}}{\pi }\int_{-a}^{a}\frac{p_{n}(s)ds}{{[(x-s{)}^{2}+z]}^{2}}-\frac{2z^{2}}{\pi }\int_{-a}^{a}\frac{p_{t}(s)(x-s)ds}{{[(x-s{)}^{2}+z]}^{2}}\\ \tau_{xz}(x,z)=-\frac{2z^{2}}{\pi }\int_{-a}^{a}\frac{p_{n}(s)(x-s{)}^{2}ds}{{[(x-s{)}^{2}+z]}^{2}}-\frac{2}{\pi }\int_{-a}^{a}\frac{p_{t}(s)(x-s{)}^{2}ds}{{[(x-s{)}^{2}+z]}^{2}} \end{array}\right.$$

where *a* is half length of the stress source; *p*_n_ and *p*_t_ are the pressure in normal and tangential directions, respectively. Then, the stress-strain history that an arbitrary point (*x*,*z*) suffers during the tool passage can be determined based on incremental thermoelastic-plastic model. This procedure can be achieved by employing different algorithms, for instance, the hybrid algorithm [23]. For the finite element method, Johnson–Cook constitutive model is a typical model applied in metal cutting simulations, expressed as follows:

(4) $$\sigma =(A+B\varepsilon^{n})(1+C\ln(\frac{\dot{\varepsilon }}{{\dot{\varepsilon }}_{0}}))(1-{\left(\frac{T-T_{r}}{T_{m}-T_{r}}\right)}^{m})$$

where *A*, *B*, *C*, *n* and *m* are material constants and stand for initial yield stress, strength coefficient, strain-rate dependency coefficient, strain work-hardening exponent and thermal softening exponent, respectively. *T*_r_ and *T*_m_ are the room temperature and melting temperature, respectively. *ε*, $\dot{\varepsilon }$ and ${\dot{\varepsilon }}_{0}$ stand for strain, strain rate and reference strain rate, respectively.

For the hardness modeling, three representative methods are always applied. Ding and Shin [18] concluded the machining-induced work hardening can be attributed to the microhardness growth due to Dynamic Phase Transformation (DPT) and Severe Plastic Deformation (SPD). The effect of SPD on microhardness was modeled based on the theories of grain refinement and dislocation density evolution. The equations are given as below:

(5) $$\Delta h_{\mathrm{DPT}}=\sum_{i=1}^{\nu }f_{i}h_{i}-h_{0}$$

(6) $$\Delta h_{\mathrm{SPD}}=k_{h}M_{t}\alpha_{0}Gb\sqrt{\rho_{\mathrm{tot}}}$$

where *f_i_* and *h_i_* are the proportion and hardness of phase *i*, respectively; *h*_0_ is the initial material hardness; *k*_h_, *M*_t_ and *α*_0_ are model constants; *G* is the shear modulus; *b* is the Burgers vector magnitude.

From the perspective of grain recrystallization, Zener–Hollomon equation can serve for the calculation of grain size as follows [15]:

(7) $$Z=\dot{\varepsilon }\exp(\frac{Q_{1}}{RT})$$

Then, the microhardness change can be evaluated using Hall–Petch equation [15]:

(8) $$\begin{array}{l} d=b_{1}Z^{m_{1}}\\ HV=C_{0}+C_{1}d^{-0.5} \end{array}$$

where *Z* is the Zener–Hollomon parameter; *Q*_1_ is the apparent activation energy for mechanical deformation; *R* is the universal gas constant; *d* is the recrystallized grain size; *b*_1_, *m*_1_, *C*_0_, *C*_1_ are model constants; *HV* is the Vickers hardness.

Umbrello and Filice [17] proposed a simple thermal model for hardness calculation concerning the formation of white layer and dark layer, which can be illustrated as follows:

(9) $$\Delta HRC_{\mathrm{quenching}}=F_{1}(\frac{67-HRC_{\mathrm{initial}}}{1030-T_{\mathrm{wlstart}}})(T-T_{\mathrm{wlstart}})$$

(10) $$\Delta HRC_{\mathrm{tempering}}=F_{2}(\frac{HRC_{\mathrm{initial}}-HRC_{\mathrm{twl}}}{T_{\mathrm{wlstart}}-T_{\mathrm{dlstart}}})(T_{\mathrm{dlstart}}-T)$$

where Δ*HRC*_quenching_ and Δ*HRC*_tempering_ are the Rockwell hardness change due to quenching process and tempering process, respectively; *F*_1_ and *F*_2_ are empirically calibrated functions; *HRC*_initial_ is the hardness of initial material; *T*_wlstart_ and *T*_dlstart_ are the austenite-start temperature (related to the formation of white layer) and tempering-start temperature (related to the formation of dark layer), respectively; *HRC*_twl_ is the fully tempered material hardness at *T*_wlstart_.

**Figure 1:**
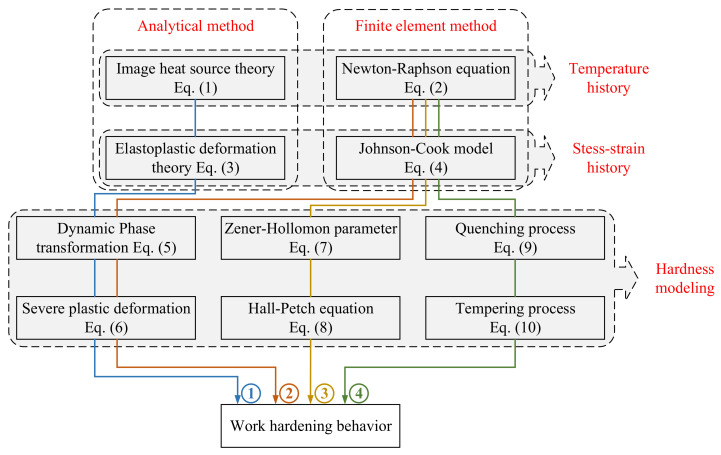
Methods for the determination of work hardening behavior in metal cutting.

With the models mentioned above, the determination of work hardening layer can be concluded as four representative routes (see Figure 1). Route 1 is a fully analytical approach developed by Zhang, Wang, Hu and Wang [19] when investigating the microhardness change of AISI stainless steel. Other routes can be clustered as hybrid approaches. The representative work of route 2 is given by Ding and Shin [18]. Route 3 is a widely used strategy employed by [16]. Route 4 is used in Ref. [17]. The advantages and disadvantages of these routes are described in Table 1.

When it comes to micromachining, some scholars applied molecular dynamics simulation for the modeling of machining-induced microhardness change [24]. This paper mainly focuses on the machining process of Inconel 718; hence, research in that field will not be further analyzed. In the paper, Route 3, which combined the grain-size-based functions (Equations (7) and (8)) and finite element simulation, was adopted to investigate the work hardening behavior for its effectiveness and operability.

## 3. Simulation and Experimental Setup

### 3.1. Simulation Setup

**Figure 2:**
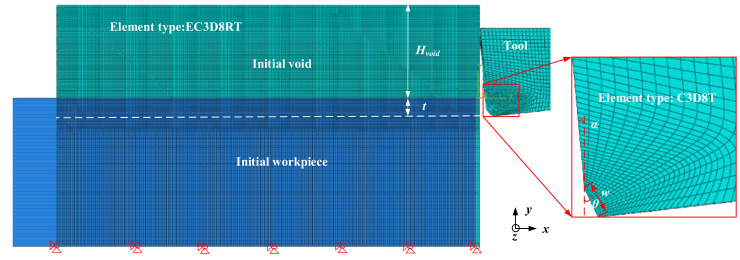
Finite element simulation setup.

A three-dimensional model was established to simulate the orthogonal cutting process using finite element method. Figure 2 illustrates the model established by ABAQUS/EXPLICIT 2020 software (Dassault Systems, Vélizy-Villacoublay, France). The CEL formulation was adopted in the model [1]. Specifically, the cutting tool was governed by Lagrangian method and the Eulerian theory was used to describe the workpiece material. In Lagrangian simulation practice, a damage layer with separation criterion was used to separate the chip from workpiece [2]. Once the distorted elements in this layer reached the separation criterion, the material stiffness was fully degraded and then the elements would be deleted from the simulation [3]. These deleted elements led to the loss of some high deformation values, as a result, the simulation accuracy decreased [4]. In order to avoid the problems of element distortion in Lagrangian theory, some other methods were created, including the Arbitrary Lagrangian Eulerian (ALE) and CEL. ALE method regarded the chip formation as the material flow around the cutting edge [5], and both the element deletion and separation criterion were not needed. However, ALE method can only be used to simulate the continuous chip formation for the absence of damage material model. Serrated chips are always observed in cutting some hard-to-machine materials (e.g., Inconel 718); therefore, the CEL method was used in this study to investigate the work hardening in machining Inconel 718.

The geometrical parameters for the simulation model are also shown in Figure 2. The initial void was established in the model, and the height of this area above the workpiece (*H*_void_) was large enough to ensure that the chip formed entirely in the Eulerian mesh. In the figure, *t* refers to uncut chip thickness (UCT), which is equivalent to feed rate in orthogonal cutting. And the assumption is *t* remains stable during the cutting process. The rake angle (*α*) and clearance angle (*β*) of the tool were −5° and 7°, respectively. *θ* and *W* were defined as chamfer angle and chamfer width. As for the boundary conditions, the workpiece maintained a constant cutting speed (*v*) along the opposite of the *x*-direction. Besides, the freedom of initial void both in the *x* and *y* direction were limited.

**Figure 3:**
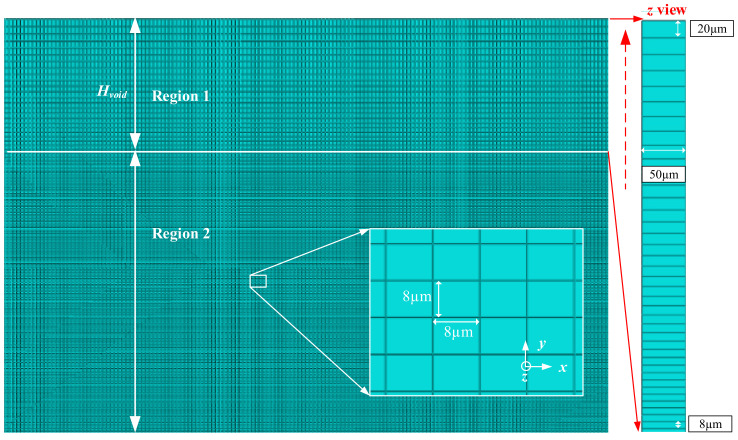
Element size layout.

It is noted that only a three-dimensional model can be used in CEL method. As a result, the simulation time is longer than other two-dimensional models using Lagrangian method. Proper selection of element size can shorten the simulation time largely. Figure 3 displays the element size in the model. The whole area of Eulerian mesh is divided into two regions. Region 1 is the void area above workpiece and the element size in the *x*-direction is 8 μm. As for the element size in the *y*-direction, it increases from 8 μm to 20 μm linearly. It should be noted that finer mesh occurs near the interface between region 1 and region 2, and coarser mesh happens in the other zones. In region 2 where the workpiece is located, the element size in the *x*- and *y*-direction is set as 8 μm × 8 μm. Besides, considering the three-dimensional model, the element size in the *z*-direction is 50 μm.

In the simulation, the workpiece material was Inconel 718 and the cutter was carbide tool. The material property was set as isotropic and the parameters are given in Table 2. The Hook law was used to define the material elastic deformation as expressed in Equation (11):

(11) σ=K⋅ε

where *K* is Young’s modulus; *σ* and *ε* refer to the equivalent flow stress and strain, respectively.

In this study, the cutting tool was set as rigid body and plastic deformation of the workpiece was governed by the Johnson-Cook (J-C) constitutive model expressed in Equation (4). The material constants of Inconel 718 are shown in Table 3.

To describe the machining of Inconel 718 more accurately, especially the formation of the serrated chip, the damage model was established. An energy-based ductile fracture criterion was used in this study and it consisted of two stages, including damage initiation and damage evolution. The damage initiation was determined by the variable *w* shown in Equation (12):

(12) $$w=\sum \frac{\Delta {\bar{\varepsilon }}^{\mathrm{pl}}}{{\bar{\varepsilon }}_{D}^{\mathrm{pl}}}$$

where $\Delta {\bar{\varepsilon }}^{\mathrm{pl}}$ is the increment of the equivalent plastic strain; ${\bar{\varepsilon }}_{D}^{\mathrm{pl}}$ is the equivalent plastic strain at damage initiation. The value of *w* increased with material deformation and the damage initiation started when it was equal to 1. The equivalent plastic strain was defined by the J-C ductile damage criteria as shown in Equation (13):

(13) $${\bar{\varepsilon }}_{D}^{\mathrm{pl}}=\left[d_{1}+d_{2}\exp\left(-d_{3}\frac{\sigma_{p}}{\sigma_{\mathrm{Mises}}}\right)\right]\cdot \left[1+d_{4}\ln\left(\frac{{\dot{\bar{\varepsilon }}}^{\mathrm{pl}}}{{\dot{\varepsilon }}_{0}}\right)\right]\cdot \left(1+d_{5}\frac{T-T_{r}}{T_{m}-T_{r}}\right)$$

where *d*_1_–*d*_5_ are material failure constants shown in Table 4; *σ*_p_ is the hydrostatic pressure; *σ*_mises_ is the Von Mises equivalent stress.

In the damage evolution, the displacement-based ductile fracture criterion was used. Equation (14) introduces the effective plastic displacement (${\bar{u}}^{\mathrm{pl}}$) using the characteristic length of the element (*L*).

(14) $${\bar{u}}^{\mathrm{pl}}=L\cdot {\bar{\varepsilon }}^{\mathrm{pl}}$$

The damage evolution was represented by the relative plastic displacement *D*. The expression of damage evolution is illustrated as follows:

(15) $$D=\frac{{\bar{u}}^{\mathrm{pl}}}{{\bar{u}}_{F}^{\mathrm{pl}}}$$

where ${\bar{u}}_{F}^{\mathrm{pl}}$ is the effective plastic displacement when material failure happens.

In fact, the parameter *D* reflects the stiffness state of the element. The material holds nondestructive stiffness with *D* = 0. *D* = 1 stands for the complete material stiffness failure, and meanwhile the failed meshed part is removed. However, the element deletion cannot happen in the CEL simulation model, and the value of the parameter *D* should be limited as 0.99.

SA sries of simulation tests were conducted to study the work hardening behavior affected by the chamfer edge. The cutting parameters and tool geometrical parameters are listed in Table 5.

### 3.2. Experimental Setup

**Figure 4:**
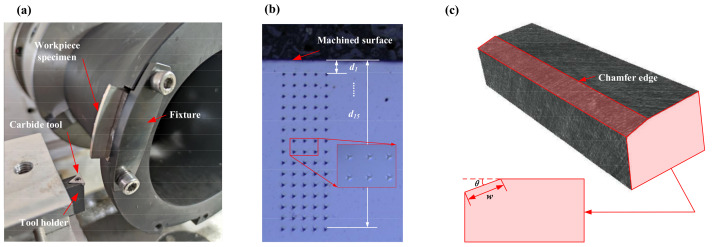
The experiment setup (**a**) The orthogonal cutting; (**b**) The nanoindentation measurement; (**c**) The edge geometry.

The comprehensive experiments were conducted to study the work hardening affected by the edge geometry and meanwhile validate the accuracy of the proposed simulation method. The cutting experiment setup is shown in Figure 4. The dry cutting experiment is expressed in Figure 4a and the turning experiment was used to achieve orthogonal cutting. The lathe type was CAK5085nzj (Shenyang Machine Tool Factory, Shenyang, China) and the triangle carbide tool was specially designed for orthogonal cutting. The rake angle and clearance angle were −5° and 7°, respectively. The cutting was dry cutting and cutting speed was 100 m/min. As for the Inconel 718 workpiece, in order to make the subsequent hardness measurement more convenient, the bar was cut into sheet specimens in advance. Figure 4b shows the hardness measurement. The NanoTest Vantage4 durometer (Micro Materials Ltd., Wrexham, UK) was used and 75 measuring points were arranged in the machined workpiece. Specifically, 15 different depths from the machined surface were selected (*d*_1_*–d*_15_) and five repeated measurements at each depth were achieved to ensure the experiment accuracy. Eventually, the hardness value at one depth was obtained through averaging the five values. Besides, the Alicona Edge Master Module was used to measure the geometrical parameters of the cutting tools, including the chamfer width and chamfer angle. The detailed information of the carbide tools is shown in Figure 4c.

## 4. Results and Discussions

### 4.1. Model Validation

#### 4.1.1. The Chip Formation

**Figure 5:**
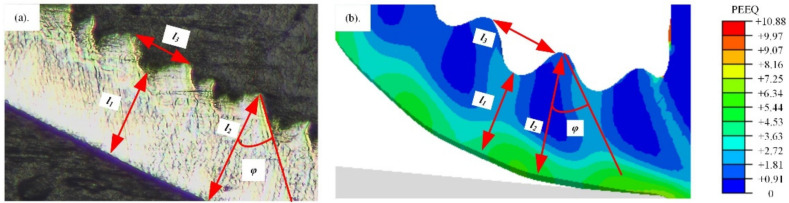
Chip formation of Inconel 718. (**a**) Chip formation in the orthogonal cutting experiment; (**b**) Chip formation in the simulation by ABAQUS.

Chip formation was an important criterion to evaluate the accuracy of the established model in the metal cutting simulation. As shown in Figure 5, the segment chip formed in machining Inconel 718. Four parameters were used to define the segment chip geometry, including *l*_1_*, l*_2_*, l*_3_ and *φ*. *l*_1_ and *l*_2_ are the minimum and maximum value of the chip thickness, respectively. *l*_3_ is the distance between the two adjacent peaks, and *φ* is the shear angle.

**Figure 6:**
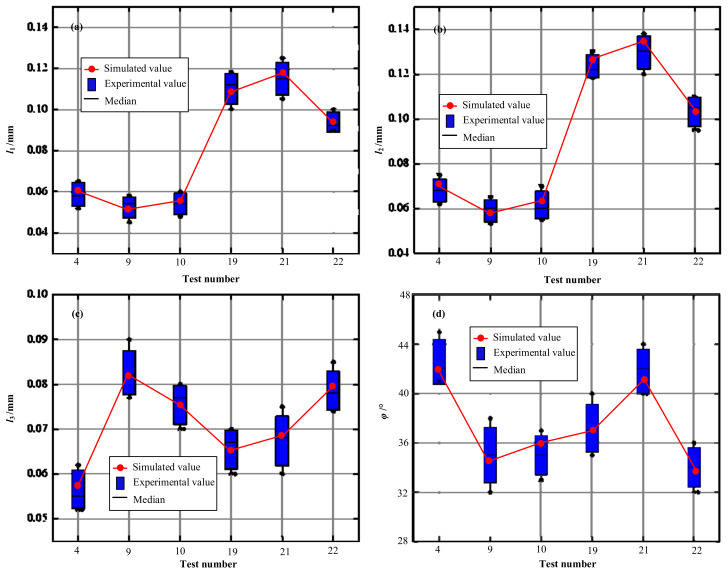
Comparison of the chip geometry between the simulated and measured results. (**a**) The results of *l*_1_; (**b**) The results of *l*_2_; (**c**) The results of *l*_3_; (**d**) The results of *φ*.

The values of these four parameters between the simulated and measured results are compared in Figure 6. The values of *l*_1_*, l*_2_*, l*_3_ and *φ* are displayed in Figure 6a–d, respectively. The errors of all parameters were relatively small, verifying the accuracy of the CEL simulation model.

#### 4.1.2. The Microhardness

**Figure 7:**
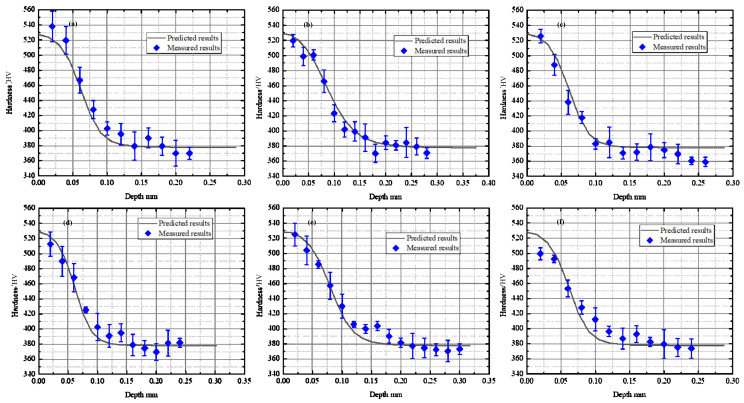
The comparison results of microhardness between the prediction and measurement. (**a**) *t* = 0.04 mm, *w* = 0.05 mm, *θ* = 25°; (**b**) *t* = 0.04 mm, *w* = 0.15 mm, *θ* = 25°; (**c**) *t* = 0.04 mm, *w* = 0.10 mm, *θ* = 15°; (**d**) *t* = 0.04 mm, *w* = 0.15 mm, *θ* = 15°; (**e**) *t* = 0.08 mm, *w* = 0.20 mm, *θ* = 15°; (**f**) *t* = 0.08 mm, *w* = 0.10 mm, *θ* = 15°.

Figure 7 illustrates the microhardness values achieved by the proposed model and experimental measurement. Various cutting and geometrical parameters were selected to ensure the reliability and applicability of the proposed model. The chamfer angle was selected as 15° and 25°, and the chamfer width was set as 0.05 mm, 0.10 mm, 0.15 mm, 0.20 mm and 0.30 mm. Besides, two different uncut chip thicknesses were considered, i.e., 0.04 mm and 0.08 mm. From the figure, it can be observed that all the measured values distributed around the predicted curves, which proved the accuracy of the proposed prediction method.

### 4.2. Microhardness under the Machined Surface

**Figure 8:**
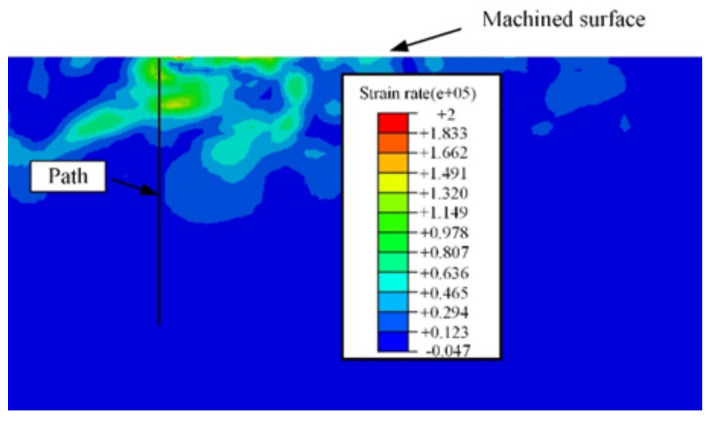
The output path setup.

The output path under the machined surface was set up as given in Figure 8. The microhardness values determined by the strain rate and temperature were extracted along this path.

**Figure 9:**
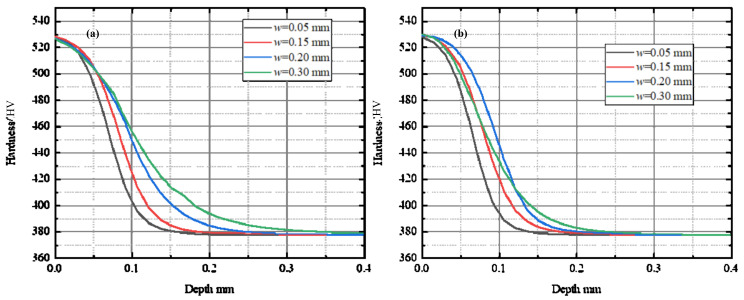
Effect of the chamfer width on work hardening. (**a**) *t* = 0.08 mm, *θ* = 35°; (**b**) *t* = 0.08 mm, *θ* = 25°.

**Figure 10:**
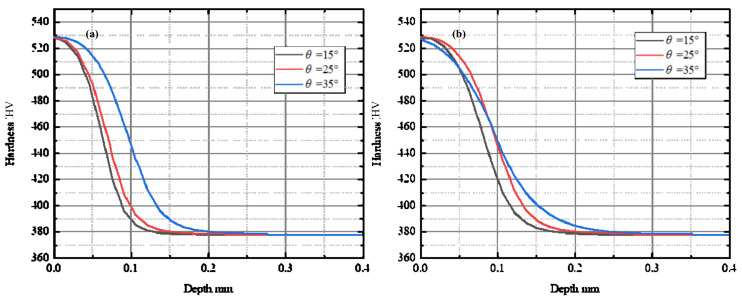
Effect of the chamfer angle on work hardening. (**a**) *t* = 0.04 mm, *w* = 0.15 mm; (**b**) *t* = 0.08 mm, *w* = 0.20 mm.

The effects of micro-geometrical parameters of the chamfered cutting tool on work hardening were illustrated as Figure 9 and Figure 10. Figure 9 shows the workpiece hardness under the machined surface with different chamfer width. Two different cutting conditions were compared in the figure. The uncut chip thickness was 0.08 mm and the chamfer angle was 35° in Figure 9a, and in Figure 9b the uncut chip thickness was 0.08 mm and the chamfer angle was 25°. From the figures, it can be seen that when chamfer width increased from 0.05 mm to 0.30 mm, the depth of the hardened layer increased. This result is consistent with the Zhuang’s research conclusion [13]. The material hardness with different chamfer angle was discussed in Figure 10. Figure 10a shows the results when the uncut chip thickness was 0.04 mm and the chamfer width was 0.15 mm, and Figure 10b shows the results of the uncut chip thickness was 0.08 mm and the chamfer width was 0.20 mm. From the figures, it can be observed that the thickness of the hardened layer increased when enlarging the chamfer angle. Consequently, from Figure 9 and Figure 10, it can be concluded that enlarging the chamfer edge of the cutting tool can enhance the work hardening behavior. However, the maximum microhardness on the machined surface almost has no change.

**Figure 11:**
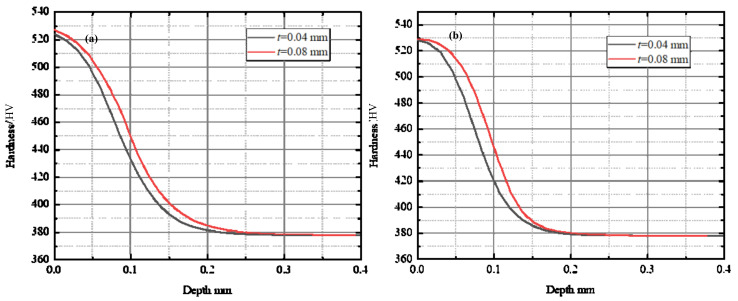
Effect of the uncut chip thickness on work hardening. (**a**) *w* = 0.20 mm, *θ* = 35°; (**b**) *w* = 0.20 mm, *θ* = 25°.

Figure 11 shows the material hardness distribution affected by the uncut chip thickness. Two different chamfer edges geometry of the cutting tool was used. In Figure 11a, *w* = 0.20 mm and *θ* = 35°; in Figure 11b, *w* = 0.20 mm and *θ* = 25°. It can be found that increasing the uncut chip thickness can contribute to the formation of the hardened layer. The same trend was also concluded in a citable work [25]. The maximum microhardness on the machined surface does not exhibit significant difference.

## 5. Conclusions

This paper aims to predict the work hardening behavior under different chamfered edge parameters when machining Inconel 718. For a deep understanding of microhardness modeling in machining, a brief literature review covering finite element method, analytical method, empirical method and hybrid methods was given firstly.

A novel method integrating finite element method and grain size-based functions was proposed to predict the microhardness in subsurface area of the machined Inconel 718. The nanoindentation experiment was conducted to measure the microhardness and the work hardening behavior of Inconel 718 existed within the hardness ranging from 560 HV to 378 HV. The predicted values were highly consistent with the experimental results, indicating the accuracy of the proposed prediction method. The work hardening behavior was affected by the chamfer edge micro-geometry. Both increasing the chamfer width and chamfer angle can enhance the work hardening. Besides, the depth of the hardened layer increased with increasing feed rate (the uncut chip thickness) in orthogonal cutting. However, the maximum microhardness on the machined surface did not exhibit significant difference.

The work hardening behavior of machining Inconel 718 was investigated considering the microstructure in this article. In the future, the microhardness introduced by phase transition will be explored.

## Figures and Tables

**Table 1:** Advantages and disadvantages of the listed routes.

Route	Advantage	Disadvantage
1	Time saving; Deep analysis of the physics.	The coding is very complicated; A lot of constants need to be quantified; Orthogonal cutting process only; Round cutting edge only; Austenitic steel only.
2	Available for both orthogonal and conventional cutting; Relatively deep analysis of the physics.	Time consuming; A lot of constants need to be quantified; Austenitic steel only.
3	Available for both orthogonal and conventional cutting; Easy to conduct; Practical effective.	Time consuming; Phase transformation is not considered.
4	Available for both orthogonal and conventional cutting; Easy to conduct; White layer and dark layer are concerned.	Time consuming; Mechanical process is not considered; Empirical functions need to be calibrated.

**Table 2:** The material properties used in the simulation.

Material	Density (ton/mm^3^)	Specific Heat (mJ/ton·°C)	Coefficient of Thermal Expansion (°C^−1^)	Young’s Modulus (MPa)	Thermal Conductivity (W/m·°C)	Poisson’s Ratio
**Inconel 718**	8.19 × 10^−9^	4.51 × 10^8^ (20 °C) 7.07 × 10^8^ (900 °C)	1.31 × 10^−5^ (20 °C) 1.71 × 10^−5^ (900 °C)	201,000 (20 °C) 173,000 (900 °C)	13.4 (20°C) 23.6 (900°C)	0.3
**Carbide**	11.9 × 10^−9^	0.203 × 10^9^	4.7 × 10^−6^	534,000	50	0.22

**Table 3:** Material constants of Inconel 718 for J-C constitutive model.

*A* (MPa)	*B* (MPa)	*C*	*n*	*m*	*T*_r_ (K)	*T*_m_ (K)
1200	1284	0.006	0.54	1.2	293	2073

**Table 4:** The constant parameters of Inconel 718 for J-C failure model.

*d*_1_	*d*_2_	*d*_3_	*d*_4_	*d*_5_
0.11	0.75	−1.45	0.04	0.89

**Table 5:** The parameters adopted in the simulation tests.

Test No.	*t* (mm)	*θ* (°)	*W* (mm)	Test No.	*t* (mm)	*θ* (°)	*W* (mm)
1	0.04	15	0.05	16	0.08	15	0.05
2	0.04	15	0.10	17	0.08	15	0.10
3	0.04	15	0.15	18	0.08	15	0.15
4	0.04	15	0.20	19	0.08	15	0.20
5	0.04	15	0.30	20	0.08	15	0.30
6	0.04	25	0.05	21	0.08	25	0.05
7	0.04	25	0.10	22	0.08	25	0.10
8	0.04	25	0.15	23	0.08	25	0.15
9	0.04	25	0.20	24	0.08	25	0.20
10	0.04	25	0.30	25	0.08	25	0.30
11	0.04	35	0.05	26	0.08	35	0.05
12	0.04	35	0.10	27	0.08	35	0.10
13	0.04	35	0.15	28	0.08	35	0.15
14	0.04	35	0.20	29	0.08	35	0.20
15	0.04	35	0.30	30	0.08	35	0.30

## Data Availability

Not applicable.

## Nomenclature

Δ*T*_1_:Δ*T*_2_	temperature rise in workpiece caused by heat source of shear zone and tool-workpiece interface, respectively
*q*_s_, *q*_r_	heat intensity of the shear zone heat source and rubbing heat source
*λ*_w_, *a*_w_	the thermal conductivity and thermal diffusivity of workpiece
*L*_s_, *L*_c_	length of the shear zone and tool-chip interface
*B*_w_	heat partition transferred into workpiece
*V*	cutting speed
*t*	uncut chip thickness
*φ*	shear angle
*C*_T_	capacitance matrix
*K*_T_	heat conduction matrix
*Q*	external and internal heat flux vector
*a*	half length of the stress source
*p*_n_, *p*_t_	pressure of the stress source in normal and tangential directions
*A*, *B*, *C*, *m*, *n*	model constants in Equation (4)
*ε*, $\dot{\varepsilon }$, ${\dot{\varepsilon }}_{0}$	strain, strain rate and the reference strain rate
*T*_m_, *T*_r_	melting temperature of workpiece, room temperature
*f_i_*, *h_i_*	the proportion and hardness of phase *i*
*k*_h_, *M*_t_, *α*_0_	model constants in Equation (6)
*G*	shear modulus
*b*	the magnitude of Burgers vector
*Z*	Zener-Hollomon parameter
*Q*_1_	the apparent activation energy for mechanical deformation
*R*	universal gas constant
*b*_1_, *m*_1_, *C*_0_, *C*_1_	model constants in Equation (8)
*HV*	Vickers hardness
Δ*HRC*_quenching_ Δ*HRC*_tempering_	the change of Rockwell hardness due to quenching processthe change of Rockwell hardness due to tempering process
*HRC*_initial_	the Rockwell hardness of initial material
*T*_wlstart_, *T*_dlstart_	austenite-start temperature and tempering-start temperature
*HRC*_twl_	the fully tempered material hardness at *T*_wlstart_
*H*_void_	the height of initial void
*α* *β*	rake angleclearance angle
*θ*, *w*	chamfer angle and chamfer width
